# How to survive the end of the future: Preppers, pathology, and the everyday crisis of insecurity

**DOI:** 10.1111/tran.12362

**Published:** 2019-12-23

**Authors:** Kezia Barker

**Affiliations:** ^1^ Department of Geography Birkbeck, University of London London UK

**Keywords:** anticipatory politics, anticipatory subjectivity, crisis, neoliberalisation of security, pathologisation, prepper

## Abstract

Emergency preparedness is a distinctive feature of contemporary anticipatory politics, yet “preppers,” a sub‐culture who prepare to survive a range of possible crisis events through practices including stockpiling and survival skill development, are subject to media ridicule and academic dismissal. If the hoarder is the symbolic deviant figure of the consumer society, the prepper is that of the security society. Such constructions of prepper pathology, however, work to reinforce the neoliberal security state. By repositioning the prepper as an amplifier of conditions of the present, what emerges is an emblematic and anticipatory figure who troubles the cracks in the security state's governing logics, exposing its social differentiation and rehearsing the inevitability of its future failures. Drawing on qualitative research on UK prepping cultures, I define prepping across three constellations of imaginative‐material practices, concerning “value,” “temporalities,” and “crisis.” I argue that prepping exposes the contradictions of infrastructural weakening alongside the networked dependencies and restricted agency felt within late modernity, challenges the expert determination of what constitutes crisis, and unveils the myth of the universality of state security protection. Living with profound crisis attunement, preppers nevertheless recuperate pleasure in material potentiality and skilful practice, in thoughtful engagement with temporalities, and in the vitality of community and meaning formed in the times and spaces in, and around, crisis.

## Abstract 2

Drawing on qualitative research on UK prepping cultures, this paper explores prepping through its imaginative‐material practices concerning “value,” “temporalities,” and “crisis.” The prepper emerges as both an emblematic and anticipatory figure who exposes the contradictions of infrastructural weakening alongside networked dependencies, challenges the expert determination of what constitutes crisis, and unveils the myth of the universality of state security protection.

[I]t just seems to be reaching a point where, even outside of the [prepper] community, people can respect or respond to these ideas, these negative ideas that something might go wrong … [M]ass society is slightly on edge, eh, and so, while they might not be in that mindset all the time, they're happy to be led there from time to time. They're happy to have a moment of zombie apocalypse. (‘James,’ prepper and regional rep for large online survival group, interview 2018)

## 1 CONDONED AND PATHOLOGICAL PREPAREDNESS

In the UK, September has become “national preparedness month,” promoted by 30days30waysUK, “the national campaign of fun preparedness games” (30days30ways, 2019). The campaign website banner, featuring a bright red ball bouncing resiliently away from an ominously dark thundercloud towards a brighter sky, tells us: “Being prepared is part of who you are, emergency preparedness is no different.” Videos and tips shared on Facebook and Twitter cover daily topics, including how to shelter in place and how to pack an emergency go‐bag. Subscribers are urged to read their local risk register and adapt their preparations to the most likely risks that could befall them.

The declaration of a climate and ecological emergency is the key demand of the campaign group Extinction Rebellion. A handbook written by the group is titled *This Is Not A Drill* (2019), adopting the language of a public announcement of an emergency situation. A Facebook group of over 4,000, inspired by Professor Jem Bendell's (2018) concept of “Deep Adaptation” to climate tragedy, share “ideas on outer and inner deep adaptation to unfolding societal breakdown.” Debate flares following the publication of a newspaper article in which a prominent Australian sustainability expert reveals his abandonment of the city – and the political process – to “prep for the apocalypse” (Israel, 2019).

Meanwhile, another crisis challenges the fraught boundary between condoned and pathological preparedness. A UK government study leaked in June 2018 outlines an “Armageddon” scenario for possible Brexit outcomes, prompting newspapers and media sites to draw attention to forms of Brexit stockpiling (Wheaton, 2018). A closed Facebook group called “the 48% preppers” is widely reported on, with the coverage swelling its membership to over 10,000 by March 2019. Members share tips on extending the life of stored products, recipes using tinned goods, and support for those who feel increasingly anxious about supply chain disruptions and civil unrest. Brexit stockpiling briefly forms the patterning of our contemporary social context in the insecurity society: *Guardian* readers are urged, tongue‐in‐cheek, to “take a night off the stockpiling to have some fun” at the Spice Girl's reunion tour (Clark, 2018).

Within debates over crisis determination and appropriate public and private anticipatory action, the “prepper” is often used as a benchmark against which to measure the rationality of responses. Wedded in media and wider cultural representations to far‐fetched visions of the apocalypse, what actually constitutes crisis for preppers varies along a spectrum of belief and practice.^1^ Ranging from short‐term personal disruptions to longer‐term societal collapse, what the experienced and anticipated crises that drive prepping share is the individual's dislocation from infrastructural services and the absence of sufficient state action to protect, rescue, or remediate. Preppers respond through a range of material practices including stockpiling food, medicine, and equipment to shelter in place (“bugging‐in”), by packing a range of mobile carries to facilitate quick escapes and enable survival on the move (“bugging‐out”), by developing survival skills, and, in some cases, by learning self‐sufficiency skills to adapt to an imagined post‐collapse environment. Their stockpiles are often hidden to guard from future theft by security forces or by the unprepared “hoards” in imaginations of future crisis. But they are also concealed to avoid the ridiculing eye of a society in which preppers are constructed as selfish or “tin foil hat wearing loonies.” However, the recent media coupling of prepping to political anxiety over Brexit and ecological anxiety over climate change – politically fraught but more socially accepted crisis concerns – has temporarily recalibrated the coordinates of difference between rational and irrational, condoned and pathological anticipatory subjectivities. This provides an opening to raise the question of why, in the face of contemporary politics supposedly geared towards securing uncertain futures, particular modes of anticipating crisis remain pathologised.

In response, this paper is not simply challenging the pathologisation of a non‐normative subjectivity situated beyond the temporal and spatial margins of the neoliberal security society, refuting or adding nuance to our constituent Other. Instead, I approach the figure of the prepper as both an emblematic and anticipatory subjectivity constituted and deeply embedded within the present. *Emblematic* in its distillation of socio‐economic inequalities in contemporary neoliberal governance that impact on our ability to secure the means to reproduce life; for preppers aim to survive this world, not just the end of it. *Anticipatory* in foreshadowing a world where apocalyptic collapse is indistinguishable from structural socio‐economic exclusion, and where the everyday citizen and not just the entrepreneur must innovate, anticipate, and dare to survive. The paper contributes to current debates in anticipatory, apocalyptic, and future geographies by examining a subjectivity embroiled in crisis and survival meaning‐making. It raises questions over the futures we examine, the way they are politicised, and their connections to the overwhelming crises of the present.

In section 3, I consider characterisations of late modernity as a neoliberal security society, and review how representations of future catastrophic risk are determining the contours of contemporary governance and the constitution of governable subjects. I highlight more limited research on the production of non‐normative security subjectivities, and consider framings of “culture bound syndrome” to open up the prepper's role as an oppositional figure by showing how they amplify and project, not just oppose, cultural norms. This is taken forward in section 3.1, “Researching prepping cultures,” which considers the historical specificity of the emergence of the prepper and provides precedents for the claim that the prepper is not simply a consequence of social context, but “an indicator, a lens, for perceiving and understanding those conditions” (Mitchell, 2001, p. 146). In outlining the research's methodological approach, I characterise the prepper through the interplay of imagination and the material across three constellations of practice central to the neoliberal security state concerning value, temporalities, and crisis. In section 4, I detail these bundles of imaginative‐material practices, contrasting the media's pathologising with the prepper's pathologised gaze. I muddy a vision of preppers' deviant material values with that of a speculative, multi‐layered, and agential material culture; an image of suspended time with the active management of multiple temporalities; and a notion of eschatological desire with the bridging of everyday and extensive crisis. In conclusion, I consider how prepping responds to inequalities and scarcity in the realm of anticipatory governance, where questions of who is protected, who can secure their own resources, who can anticipate, and who survives mirrors and projects inequalities in the capitalist system.

## 2 EMERGENCY GOVERNANCE AND PATHOLOGICAL SUBJECTIVITY IN NEOLIBERAL SOCIETY

The UK prepper emerges in the socio‐political context of what Amin (2012) describes as a turn towards the “security” or “emergency state” (Dillon, 2008; Lakoff, 2007). Several prominent scholars have offered diagnoses of the contemporary condition of late modernity, in which concerns over catastrophic futures and issues of risk and insecurity permeate society and form a central way in which we are governed, “distinctively writing the contours of [our] world” (Amoore, 2013, p. 7). Beck's (1998) characterisation of the period is of a “World Risk Society,” in which catastrophic, incalculable, and uninsurable risks proliferate out of control; Bauman (2007) names the “liquid fear” of late modernity; Amin describes “the slow creep of fear, anxiety and watchfulness into even the most intimate of spaces, as a sense of the world and home at risk takes root” (2012, p. 13). Crisis anxiety and a generalised insecurity emerge as a structure of feeling, an affective economy of fear that mediates how life is lived and thought (Anderson, 2014). In the work of Žižek (2010) and Swyngedouw (2010), the end of the future through catastrophe becomes folded into the present, hollowing out the political sphere and, in demanding only technological solutions within the existing political frame, prevents any form of meaningful change. Expert anticipatory knowledges justify the development of ever‐new modes of intervening in the present to manage, control, reduce, or prevent possible futures (Anderson, 2010). For this era of “post‐politics,” no alternatives to the capitalist present can be imagined, the promise of the future as progress is forestalled, while our capacity to wreak environmental change continues to be imprinted on the geological record (Berardi, 2011).

This body of work has attended extensively to institutional formations and practices of power, with fewer empirically informed explorations of lay public modes of knowing, experiencing, and acting to secure negative futures; what we might understand as “anticipatory subjectivities.” In the context of anticipatory politics, Amoore asks: “what forms of subjectivity, what types of population, are brought into being and made amenable to governance…?” (2013, p. 6). Building on Rose's (2006) work on “biological citizenship,” research on the subjectivising dimensions of security governance includes attention to the production and performance of “resilient subjectivities” (Cloke & Conradson, 2018) and “biosecure citizenships” (Barker, 2010). Concepts of risk and security are seen to construct norms of behaviour, understandings of safe or risky conduct through which individuals voluntarily self‐regulate. Work has traced how neoliberal security subjects are produced through the commodification, individualisation, and privatisation of insecurity within gated communities, private security firms, and self‐defence training (Ellin, 1997). Bauman's (2007) citizen of the liquid modern society is a fearful subject; Lasch (1984) describes the contours of the “minimal self.” The normalised subject is resilient, prepared, self‐motivated (and self‐financing), but also suspicious and fearful. Widespread anxiety alongside inertia has been diagnosed as symptomatic of the subject in late modern risk societies.

This exploration of subject formations made amenable to governance (Amoore, 2013) has eclipsed attention to non‐normative, marginal/ised, pathologised, or deviant security subjectivities. As Foucault's (1964) work demonstrates, the need to pathologise emerges in particular contexts as part of the production of the boundaries of community and normalised governable subjects, playing a role in policing the specificity of “healthy” and “diseased” versions of societal configurations (Lepselter, 2011). Hysteria (Foucault, 1964), fugues (Hacking, 1998), suicide (Durkheim, 1897/1952), neurasthenia (“urban modernity disorientation”; Simmel, 1903), and hoarding (Bennett, 2012) have all been explored as culturally contextual social anxieties, highlighting the social specificities and contradictions through which specific pathologies of the present emerge. However, rather than being understood as operating outside of societal formations or opposite the norm, something more significant can be extracted from these accounts of “culture bound syndromes.” The pathologised subjectivity can be grasped not only as constituted by social context, a product of social currents and concerns, but also, due to their sensitivities or attunements, as a lens that magnifies the conditions of the present (Bennett, 2012). If the hoarder is the symbolic deviant figure of the consumer society, then the prepper is that of the neoliberal security society, who expresses, amplifies, exposes, and troubles its contradictory currents.

### 2.1 Researching prepping cultures

OpSec^2^ is always going to be your enemy with this subculture. Like counting ghosts. (Private dm chat with ‘Craig,’ leader of large online prepping group, 2019)

So for all the talk of the grey man,^3^ the grey women is so much better at it she goes completely unrecorded?

And so endeth my lesson LOL. (Female prepper in open conversation with author in social media group, 2018)

Given media interest in the malevolent cultural figure that the prepper occupies, it is a surprisingly understudied subjectivity. This is in no small part due to the difficulties of conducting research on a sub‐culture for whom secrecy is paramount to their core practices (Mitchell, 2001), who have subversive humour and discursive legitimacy practices built in response to media and cultural ridicule (Campbell et al., 2019). But it is also related to their perception as a deviant figure at the *margins* of the capitalist order. This perception of marginality is written into any hesitantly compiled biography of prepping, which, understood to have emanated from the USA as the successor to the anti‐government libertarian survivalism that thrived from the 1970s to the early 2000s (Lamy, 1996; Mitchell, 2001; Myers, 1982; Wojcik, 1997), is linked in the popular imagination to a series of infamous cult and domestic terrorism incidents (Ruby Ridge, Waco, Oklahoma). Scholars posit a distinct break between prepping and survivalism (Huddleston, 2016; Mills, 2019, 2018). The latter in its representation as a home for antisocial recluses, political extremism, white supremacy, obsessive apocalyptic millenarianism, and outlandish conspiracy theories, however, inflects wider cultural perceptions of prepping. This characterisation is perpetuated through popular programmes with large audiences, such as the American reality TV series *Doomsday Preppers* (Kelly, 2016).

Mitchell's longitudinal ethnographic study can be credited for repositioning the survivalist within the academic gaze as a symbolic subject of late modernity, “a consequence of modern times and a means by which modernity may be understood” (2001, p. 5). Mitchell's intervention is also methodological. While Lamy argues that the complexity of the practice and belief system of his extreme version of the survivalist “can be uncovered in the literature, media, and industry of survivalism” (1996, p. 70), Mitchell criticises academic approaches that “forgo the observation of lived survivalism in favour of passive texts” (2001, p. 18). Taking up the mantel of in‐depth, multi‐method research, recent accounts of prepping as a 21st‐century phenomenon in the USA have built on this portrayal, positioning prepping as a product of mainstream‐right American politics (Clinton, 2017; Huddleston, 2016; Mills, 2019, 2018; Sims & Grigsby, 2019).

From these US roots, prepping has become a global phenomenon, with national prepping cultures in France (Vidal, 2015), Sweden (Rahm, 2013), and South Africa (Senekal, 2014) subject to academic analysis. In the UK, figureheads of the prepping scene describe the formation of small local groups in the early 2000s scattered nationally, with members invited via personal endorsement, and some connecting through early‐use internet chat‐boards. The scene grew from these early pioneers, enabled by online social media, and gained exposure and notoriety from the American reality TV series *Doomsday Preppers* which aired between 2011 and 2014 on the National Geographic Channel, followed by the UK versions *Preppers UK: Surviving Armageddon* and *Preppers UK 2*. Real and anticipated events, including Y2K, 9/11, and later the 2007/8 financial crisis and the 2011 London riots, were key focusing events. Prepping in the UK, however, connects to a longer history of citizen responsibilisation and individualisation in disaster preparedness, stretching back to the imperative to tend gardens and stock pantries during the Second World War. In this paper I hope to build a picture that begins to answer the question of why preppers have emerged in the UK with force when they have, by contextualising prepping as a phenomenon of the present, not a sub‐culture left in its wake.

My account draws on material produced through an ongoing research project, including 18 in‐depth interviews with UK preppers (recruited from online groups, personal endorsements, Mumsnet, and permaculture forums); observant participation and “campfire interviews” at over a dozen prepping‐related meets and survival training weekends; social media analysis of five online prepping groups in 2018–2019 and 15 prepper‐related YouTube video channels, blogs, individually hosted Facebook pages, and Instagram accounts. Four key informants were followed across these different online and offline spaces, with one participating in multiple interviews totalling over 12 hr.^4^ The analysis of media representations of the UK prepper is derived from reviewing 270 articles from UK‐based newspapers retrieved using the search terms “prepper” and “survivalism” between 2012 and 2018, with additional terms of “panic buying,” “hoard*,” and “stockpil*” in 2018, filtering for relevance. Three documentaries were analysed; *Preppers UK: Surviving Armageddon* (National Geographic Channel [NGC], 2012), *Preppers 2* (National Geographic Channel [NGC], 2013), which were produced by National Geographic Channel; and the short documentary *Apocalypse how?*, produced for the *Guardian* newspaper (Sprenger & Healey, 2017).

A stereotypical prepper will be… Yeah, none of us are stereotypical. I've seen preppers that have got three tins of baked beans and a rucksack – that's it, and they think … they're done. I have seen people that have got close to £15,000 worth of equipment, preps and everything, and they are … secured to a point where they could be self‐sufficient for over two years. These are people that I know. (‘Craig,’ interview 2019)

Prepping in the UK is not a monolithic culture and the attitudes of the people I have met defy easy political classification. Instead, it is best understood as a spectrum where in online and offline spaces, different group dynamics, issue foci, and performative practices can be discerned; even the embrace and terminology of prepping is not agreed (Campbell et al., 2019). In some iterations, prepping merges happily with bushcraft and wild camping; in others, a more overt militarised survivalism is exhibited and the bushcraft hipster is deplored; and more universally, the terms “armchair prepper” and “doomsday prepper” function as distancing devices. The differing “body language” of online prepping communities alters their demographics.^5^ In some groups, efforts have been made to address the gender imbalance, and women‐only groups have formed. In contrast, the overtly white, heteronormative, and nationalistic tenor of many prepping groups is rarely problematised.

How then can we distinguish, characterise, and understand the prepper? Campbell et al. (2019) argue that being a prepper is an attitudinal state; Mills (2019, 2018) attends to the core appeals and anxieties that motivate people to become preppers, arguing that it is wedded to political partisanship; Kabel and Chmidling (2014) define preppers through a rugged individualistic personality type; Kelly (2016) suggests that the prepper is an iteration of masculinity in crisis; Clinton (2017) argues that prepping manifests a bunker mentality orientated towards preserving middle‐class heterosexual privilege. In contrast to these approaches, I argue that the subjectivity of the UK prepper across its spectrum of expression and instability of categories should be grasped through its unique constellation of “storied” material practices – material expressions of future imaginations (Mitchell, 2001) – which are performed in relation to the crisis coordinates of the neoliberal capitalist and weak democratic present. In the following, I argue for attention to how value is mobilised, how futures are circulated, and how crisis is defined, as critical not only in characterising prepping, but also as key formations in the intersection between neoliberal capitalism and security governance. Across these themes, I knit together the pathologising and pathologised gaze to approach the prepper as both an emblematic and anticipatory figure who provides an optic to magnify, and glimpse beyond, the tyranny of the present.

## 3 THE MATERIAL IMAGINATIONS OF SURVIVAL

### 3.1 Value

There are few things preppers love more than their survival equipment, known as their preps. (NGC, 2012, p. 39:36)

In media representations, prepping is a material obsession struck through with epistemological and aesthetic ambiguity: either pathologically over‐valuing, irrationally mixing, or fetishising power in objects. The staple spectacle is that of the prepper standing defiantly in front of a visual inventory of their amassed items. Garage shelves or kitchen cupboards are shown tightly packed with tins and jars, rolls upon rolls of toilet paper, walls of tools and weaponry (Moshakis, 2018). Despite being obsessively well organised, the muddling together of high‐ and low‐value objects distorts “normal” object relations, suggesting that “the strict rules for classifying and comprehending phenomena no longer apply” (Plotz, 2005, p. 118). On the one hand, preppers are seen to be over‐investing in expensive equipment for specific tasks: the NBC suit, the adapted Land Rover, the generator, the gas mask, the water filtration system; specialised items with questionable use‐value when coupled only to far‐fetched visions of catastrophic future events (NGC, 2012). On the other hand, the comic mis‐valuing of everyday low‐value items is implied through the media gaze lingering on endless cans of beans, pot‐noodles, gravy granules, toilet paper, spam: everyday items with low present or speculative monetary value, and with no cultural capital. As these items mingle incoherently, an idealised order of material relations and transparent system of attributing value is contravened. Materiality emerges as a trope mobilised to construct prepper pathologisation by implying an abhorrent subversion of value (Herring, 2011).

Turning to the prepper life‐world beyond these media representations, we do find multiple, layered, and transient material values. Rather than chaotic and incoherent hoarding, however, this multiplicity signals a system of value based on material flexibility and potentiality (Bennett, 2012). Prepper social media is filled with photos of equipment, neatly organised in a “knolling” style and often labelled; and in endless YouTube videos preppers unpack, display, and talk through their equipment. Particular items and brands are highly prized, such as certain ex‐military kit and products from well‐regarded independent makers, and lively debates are enjoyed on the best backpack/sleep system/billy‐can/stove/torch/knife/axe. Getting a good bargain is well respected, and gift exchange adds value and prestige to items, particularly if from noted community figures:

I try, where possible, just to really enjoy the objects themselves and to try and have a story around them, to know the person who made it, or to have the story about who I traded it with … (‘James,’ interview 2018)

Crucially, the value of these different objects is not sitting in suspension even though their “crisis potentiality” is yet to be invoked. Their value is retrieved and utilised every day: topping up weekly food, on the weekend camping trip, in the sticking plaster over the small everyday crisis. These objects are not held in untouchable revere but are adapted, tinkered with, and improved, through practice, play, or performance. “James” spoke about the pleasure of being involved in this “maker community” (Nguyen, 2018):

You know, all of a sudden … you're surrounded by a community of really creative people, who are interested in solving problems, they're good at it, eh, and, yeah, some of them [are] just making beautiful stuff [laughing]. (Interview 2018)

There is enjoyment, social recognition, a sense of community, prestige from peers, and validation through the identity they help secure as a resourceful person or sensible parent, all produced through and retrieved in the present from these materials (Kelly, 2016).

However, the material culture of prepping also stores potential value that can be activated suddenly and when needed in a future emergency. In these scenarios, present value would detach from objects as the cultural, legal, and physical apparatus for valuing items collapses. A prized prepping status symbol that implies know‐how in the present might in future mark you out as a target and put your stores at risk from robbery; “grey man” clothing and innocuous mobile carry items are kept for this eventuality:

[I]n those kind of situations because you need to be the grey‐man, and with something like [gesturing to a camouflage backpack] getting from A to B, I'm going to stand out like a turd on a pool table. (‘Ian,’ recent member of online prepping group, interview 2018)

Value is then imaginatively reattached through the specificity of assumptions about future scenarios and the moral and legal worlds they trace (Kabel & Chmidling, 2014). Knives and other self‐defence equipment that are illegal now may change status in the permanent breakdown of society (WROL)^6^ and procure advantage in the ensuing violence. What is prized above all is an open, speculative material potentiality, in which objects are physically and imaginatively flexible and adaptive to different contexts, supporting general readiness for all sorts of eventualities (Huddleston, 2016), as “Ian” described:

I now look at things and think, well, that would be a handy material for this, that or other; and that, I could turn into that, and this would be a good thing, and that … I'm looking at things very differently now. I'm seeing two or three uses in one item and [pause] its [pause]. Resourcefulness. I'm looking at things that can be done to [pause]. Do you know what I'm kind of getting at?

Yeah

I see things differently. (Interview 2018)

Bunkers, the classic imaginary architecture of the pathologised prepper, do not offer this flexibility for different scenarios:

We have an ongoing joke. It's like kind of, people mention bunkers, ‘Shhh, don't talk about the bunkers!’ But none of us have them because they're … not adapting [sic] at all. Bunkers are only good for like two out of possibly like eight or nine situations. (‘Craig’ interview 2018) 7

Ordinary, everyday objects *can* be mined for alternative uses. Condoms can carry water, tampons and Vaseline are useful for fire lighting, and tampons with applicators can be used to filter water, like a drinking straw (“all these guys actually carry tampons in their survival kit, but do they think of their daughters' need for sanitary wear in SHTF^8^ situations?”; “Claire,” interview 2018). Across a broad range of forms of consumption, items are interrogated for their ability to be attainable, adaptable, replicated, or (multi)functional in future scenarios of infrastructural disconnection.

In the “testing grounds” of the crisis (Kelly, 2016), however, survival equipment may not perform as and when intended, or it may be stolen or lost, so preppers should master at least three different ways of lighting a fire, purifying water, and making shelter; “three is two, two is one, one is none” is a common prepping adage (then there's the 5‐5‐5 challenge: can you provide each in five minutes? With your dominant arm injured?). Crucially, material potentiality is not innate and atomised but relational and performative, and needs skill to extract, activate, and channel its value. This emerges in a reverence for practice, experimentation, use, and training, the dismissal of armchair preppers and equipment junkies, and supports a small industry of bushcraft and survival skill training courses and endless YouTube videos of skill demonstrations (Huddleston, 2016; on “folk preparedness pedagogies,” see Nguyen, 2018). “James” placed this emphasis on learning practical skills at the heart of his definition of preppers: “So, the learning how to make stuff, learning how to plant stuff, just learning, learning, learning, it's just … the kind of people I guess a lot of preppers are” (interview 2018); “Ian” echoed this language as he reflected on his enjoyment of learning through tinkering: “I like to learn. I'm very hands‐on. I'm always fiddling around with something, and I always try and do as much as I can” (interview 2018). Skill and practical know‐how is demonstrated through mining and bringing out the value of mundane items; understanding the value of well‐made items; and recovering the value of unloved items.

By attending to object potentiality, to what a thing could become if embedded in a different set of relations – when decoupled from their usual assemblages or part of a different legal and moral universe – prepping yearns for unstructured potential, for alternatives, for creative roles in determining value and culture (Lepselter, 2011; Mitchell, 2001). In this way the prepper interpolates, expands, and distorts a version of the entrepreneur by speculating, adapting, and generating alternative forms of value. Preppers activate the desire, captured in the liquidity of the stockpile, to act freely and at will (Peebles, 2014; Tsing, 2015), but also to be challenged and measured, tested and valued in themselves (Mitchell, 2001).

Prepping's investing of value in material potentiality, flexibility, and mastery, however, also exposes a deeply held anxiety of network dependency in late modernity's hyper‐interconnected society, alongside more widespread concerns over the capacity of weakened infrastructure to secure the reproduction of modern life. The stuff of prepping can be understood as a “shadow infrastructure,” curated to enable material autonomy in the crisis space beyond collapse. Prepping glimpses towards a possible future embedded in the present where the infrastructural provision of our metabolic requirements has been undermined and privatised to the point of exclusion. In the remaining spaces and fragments, the mastery of resources and value, the control of the means of production as a structuring force of the social is distorted into the basis of survival.

### 3.2 Temporalities

‘neoliberal time’ … gives rise to … temporal practices of suspending the self, suspending hope for change, and suspending an unfolding future, a kind of withdrawal of time… (Baraitser, 2017, pp. 161–162)

In media representations of prepping, deviance emerges through tropes of temporal suspension: both of value circulation in the petrified stockpile, and the associated social and political suspension and atomisation of the prepper. The prepper's rows of tins, bottles, sealed tubs, and wall‐mounted armoury are made to look obscene as they sit, frozen, waiting for crisis to animate and activate their value (Dixon, 2012; Sawa, 2018). This is not so much “matter out of place” (Douglas, 1996), but matter out of time, condemned never to transition to either use value or waste: outside of consumption, exchange, and discard. The folly of the suspended stockpile comes to represent a wasted life on hold. The prepper is portrayed as living outside the flow of time, as if their attachment to apocalyptic futures folded into the present produces a thickening or coagulation of present time in a way that prevents time's passage (Baraitser, 2017). This suspended time seems to cling to and restrict the prepper, who remains orientated to the future yet living in a time “that will not unfold” (Baraitser, 2017, p. 5), poised on the brink of an imaginative future that will never come. In contrast to the hypnotic allure of apocalyptic end time and abrupt rupture, despite the busyness of their preparations, prepping time seems dull, empty, suspended, petrified. This congealed future‐present of the pathologised prepper epitomises the post‐political continuous present and associated social atomisation, restricted agency, and political impotency of late modernity (Bauman, 2007; Lasch, 1984). Perhaps it provides us with some reassurance that against this caricature, our own lives are more open, agential, and sociable than they may feel.

In contrast to this picture of crisis futures coagulating in the present causing a suspension of time, I argue that prepping demands active engagement with temporal circulations and multiplicities, and the interrogation of endings, dependencies, and obsolescence. Rather than living passively with the end of the future, futures as endings and openings adhere in prepping material practices. They are sensed, produced, recorded, shared, played with, crafted, embellished, picked over, moralised, and circulated. The prepper's embrace of a state of ongoing readiness – of perpetual contingency in contrast to contingent dependency – demands an agential engagement with temporalities (Kabel & Chmidling, 2014).

On a social media group, a prepper posts photos of the putrid remains of cans buried in their garage eight years ago, supposedly in an airtight box. Beneath the photo, sympathy, criticism, jokes, and tips for how they could better manage their preps by circulating them through their everyday stores dominate the comments. *Successful* home storage of volumes of food demands intimacy and knowledgeability about the temporal life of the food product and its packaging. Preppers become acutely aware and astute managers of the temporalities of food as they practice different modes of food autonomy, in ways that contrast with the passive role of the contingent consumer dependent on a just‐in‐time food provisioning economy (Vidal, 2015). In detailing the difference that being a prepper makes to her everyday patterns of storing, shopping, cooking, and eating food, “Claire” described how she tore out the utility room next to her kitchen and adapted this as a storeroom, improving the flow between her prepping stores and everyday cupboards (interview 2018). Tips circulating in prepper social media include shelving designs that allow cans to be stacked with the most recent use‐by date accessible first; others use spreadsheets to keep track of these treacherous temporalities. Modes of long‐term food storage such as dehydration, vacuum packing, bottling, and pickling are all highly regarded skills. Yet both processed food (such as tins or MREs^9^) and home‐grown and preserved food have status, enjoyed in the present as efficient and honest or authentic and celebratory, and layered with future value according to the specificities of the imagined crisis within which they could perform.

Forms of temporal circulation extend to the social milieu of prepping and its merging of online and offline spaces, future scenarios, and historical re‐enactments, stories of war that maintain a callous grip on the present, and speculative futures that will plunge us back into our pre‐electrified past. Preppers are networked individuals, “members of multiple and technologically mediated networks of affiliation” (Amin, 2012, p. 13), across Facebook, YouTube, Instagram, and Twitter, including open as well as closed, invitation‐only groups and chat‐rooms. Groups form, have peaks and troughs of energy, split; rival groups are formed, taking and shedding ideas and practices. Conversations started in public forums drift into direct messaging; posts link between YouTube, newspaper articles, and blog entries; the hosts of livestreams on social media greet viewers in real time as they tune in to watch, like, and comment. While the online world is the dominant ongoing form of sociability, there are regular events for preppers to meet directly; at annual group camps, training weekends, or more informal or private meets restricted to closer acquaintances, as well as public shows and expos in overlapping fields of interest (including bushcraft, wild camping, knife, military, and re‐enactment). Online and offline spaces are not hermeneutically sealed. At one annual camp, four “celebrity” preppers filmed for their individual YouTube channels. The video links circulated via Instagram and connected one to the other in the comments, blurring the temporalities and boundaries of participation, and drawing the events into a storytelling genre (Mitchell, 2001). This online and offline sociability, identity‐building, and community participation – a prefigurative form of “disaster solidarity” (Solnit, 2009) – was frequently cited in interviews as a driver behind participation (see also Aldousari, 2015; Kabel & Chmidling, 2014).

Through the active management and attentive care of future temporalities circulating through the present, the prepper invokes a desire for freedom (Tsing, 2015), as if through the activation of distinct temporalities a form of autonomy can be realised and a creative role in an open future can be forged (Mitchell, 2001). Preppers are, however, represented as a risk figure with the potential to cause a crisis of circulation, if their coagulated present were to block the arteries of commercial exchange and prevent value from being generated, as neoliberal temporalities dictate.^10^ Together, these opposing stories amplify the neoliberal coupling of temporality and value generation, and gesture towards a possible world sensed by preppers where the ability to anticipate and speculate, to capture and circulate value in crisis becomes a basic means to survival. Nevertheless, prepping tinkers with an alternative materiality and temporal frame that exists outside a liberal way of life (Wakefield, 2018); which, in Baraitser's words, has “a tangential relation to those that characterise ‘the capitalist everyday,’ thereby stilling, even if they don't manage to disrupt, modes of production based on utility or exchange” (2017, p. 2). It does so in ways that can be productive of skill, identity, community, and a sense of security.

### 3.3 Crisis

The common misconceptions: one, we're all waiting for something to happen, two, that we will be happy when it does. (‘Craig,’ interview 2018)

The prepper is shown embedded in a world of televised risk. News images of catastrophic flooding, urban rioting, forest fires, and hurricanes cut across the screen; the picture flickers and jolts, the colours drain to black, white, red (NGC, 2012, 2013). The clash and scream of the “meep” – the media term for background music that accentuates a scene's intensity (Herring, 2011) – accompanies this supposed evidencing of insecurity, crisis, disaster. But then the camera cuts to mutely coloured shots of safe suburbia. The prepper walks purposefully (with an air of superiority) up an ordinary high street with shoppers milling about, and the soundtrack tellingly shifts to the delicate tune of whimsical chimes (NGC, 2012). The boundaries between this aesthetic of crisis and normalcy are teasingly overlapped for comical effect. The prepper in their camouflage gear is placed in scenes of domesticity (floral wallpaper is ideal). The tactical Land Rover is shot against the background of new‐build suburbia with twitching net curtains. This juxtaposition of safe, boring suburbia and apocalyptic fears, rather than being uncanny, produces an effect of ridicule.

Prepper pathology in media portrayals is strongly associated with the emotions of eschatological desire or longing. Preppers are shown to be eagerly awaiting the coming apocalypse, a framing mirrored in some academic analyses (Foster, 2014; Kelly, 2016). “I'm prepping for civil unrest!,” “I'm prepping for a new dark age!,” the preppers from *Preppers UK: Surviving Armageddon* enthusiastically exclaim (NGC, 2012). “Inside Britain's Armageddon houses!” the *MailOnline* invites (Dixon, 2012). “Apocalypse soon,” promises *The Times* (Marsh, 2015). This obsession is evidenced through the macabre objects filling their homes, everyday reminders of future catastrophe in the preppers' lifeworld. Alongside the suspended temporality of the stockpile discussed above, then, the representation of the prepper relies on a further temporal frame. Preppers are portrayed as caught in teleological time, always focused on a future catastrophe beyond the present in a way that falsely forecloses the potentiality of the future. Wedded to change only through dramatic rupture (Badiou, 2006), the possibility of positive or incremental change is negated.

Rather than oppose this picture of a blurring between normalcy and crisis, I wish to reinforce and expand its significance. Recent academic work has begun to reappraise the place and form of catastrophe in prepper lifeworlds. Mills (2018) argues that US preppers reveal a non‐apocalyptic, imprecise, and negative view of collapse, breaking with other academic framings (Kabel & Chmidling, 2014). My research corroborates the ordinariness of the crisis focus in the discourse of preppers, yet I would go further in arguing that the endemic personal crises of everyday life, the impacts of regimes of austerity and dispossession, the limits of the state in terms of the production of vulnerability and absence of appropriate welfare support, feature as a significant driver of prepping in the UK. Preppers often point to their own experiences of personal crisis and precarity as motivation for their prepping. Expressions of individual insecurity include financial difficulty, job loss, concerns over physical and mental health, inappropriate housing or homelessness, house fire, the cessation of welfare payments or transition onto universal credit, and particularly, the mental health challenges experienced after leaving the army. As “Craig” put it in explaining the motivations of preppers: “everyone has had something bad happen” (interview 2018). These are not apocalyptic threats from outside but ones sensed to be already here or inescapable, produced through unevenly distributed vulnerabilities, a generalised precarity, the everyday endemic “ordinary” crises at the heart of the neoliberal security society. In discussing how his experience of poor health contributed to his awakening into prepping, “James” explained:

I was always in a sense of, eh, a heightened sense of fight or flight, always waiting to have the shit kicked out of me, you know, by this [medical] condition, and I suppose, at that point, I found myself really having to clutch at, em, structured routines, carrying objects, really getting into the prepping mindset. But I wasn't preparing for a nuclear holocaust, I was preparing for my own neurological holocaust. (Interview 2018)

On social media discussion boards there are multiple references to the prepping stockpile as a “personal food bank,” of being able to fall back on your supplies in periods of unemployment, illness, or the feared six‐week wait for universal credit. “Liam,” a dedicated prepper and father of a young family of five, keeps several large barrels of rice, flour and pasta, alongside tins, in the corner of a bedroom for lack of space. He estimates that with rationing it could feed his family for six months. He is unemployed when we speak, so thankful for his food stockpile, which acts like a buffer or reservoir, allowing him to manage the ebb and flow of finances. The car factory where he formerly worked is the main employer in his town “and not a lot else”; in the past he has twice given away his food preps to colleagues who lost their jobs (interview 2018). Such experiences contribute to a loss of faith in the state for provision, assurance, and relief in crisis. They are wrapped into an unspecific ill‐ease, a sense that the mounting tensions exerted on the present cannot continue to be absorbed without rupture. They are related as a wake‐up call, a test of weaknesses prompting a determination to be “better prepared” next time, and generate sensitivity to other larger scale crises:

I did have to make sure I knew where my safe places were from a medical perspective, but the repercussions were that I was just becoming hyper‐sensitive to potential threats, potential problems that I might have to solve for my family. (‘James,’ interview 2018)

Through these personal awakening experiences, preppers become preternaturally crisis‐attuned, hyper‐sensitive to insecure futures folded into the present, into the everyday (Bennett, 2012).

Campbell et al. query preppers' use of the ordinary crisis and references to common sense preparedness as a strategy “to enlist cognition as a superior form of legitimacy, and to cast themselves as boring, mundane, with shared commonalities with many mainstream welfare concerns, and thereby dispelling the stigma of the ‘big catastrophe’” (2019, p. 9). The big catastrophe – whether SHTF or TEOTWAWKI^11^ – certainly circulates as a significant theme in prepping discourse. Yet it is the way it is discursively merged with everyday crisis that is significant. As preppers blur typical distinctions between the personal crisis and widespread catastrophe, what holds them together then is the *form* of crisis as infrastructural dislocation and the *material texture* of individual precautionary practices taken in response. The everyday crisis is expressed as an abrupt disembedding, an atomisation from infrastructural networks and welfare support structures, which mirrors the imagination of widespread social and infrastructural collapse.

This bundling together of crises of different type and extent can be done in humorous and flippant ways. Just as SHTF denotes a range of events from the personal to the widespread (“I guess this is my personal SHTF”), reference to the zombie apocalypse not only acknowledges and subverts media ridicule; it also draws on the enveloping nature that this particular imagined crisis provides as a focus for preps. As Huddlestone details, “the preparations and skills necessary to weather these events are the same … The need for food, water, and safety are paramount” (2016, p. 241). If you are prepared for a zombie apocalypse, you are prepared for anything (see also Clinton, 2017).

The deviant figure of the doomsday prepper focused expectantly on the apocalypse works to naturalise the indefinite continuity of capitalism by solidifying the idea that we cannot – and should not – imagine an end to the present; it would be mad to do so.^12^ Construed as overreacting to media hype, this also works to excuse wider inaction and apathy in the face of the economic and environmental crises of the present. By constructing individual preparedness responses motivated by self‐identified crises as pathological, the prepper is shown to overreach their agency and capacity for determining vulnerability. However, rather than simply bringing exceptional crises into spaces of normalcy, the prepper refuses the construction of the normal crisis as exceptional. While this challenges the expert monopoly on naming crises and determining acceptable levels of risk and response, it also foreshadows a future‐present where catastrophe and crisis have become the baseline of our everyday and the objects around which capital, identity, community, and politics are produced.

## 4 CONCLUSIONS

[I]t is time to wake up from the dream of safety those in power want us to dream. (Selco, 2019)

[W]hat I'm saying to you is, *they're* [the UK government] okay, *they're* catered for – we've got nothing. We've got to look after ourselves. So, no, I don't trust them. I don't … believe what they say. And when they give you these public [preparedness advice], em, oh, ‘This situation is going to happen, and this is what you must do,’ really, you might as well just stick your head in a cornflakes packet for what half of these things are going to do. (‘Ian,’ interview 2018)

What are prepping cultures, through their heightened sensitivity to the patterning of future crisis and its foldings into the everyday, illuminating about the particular currency of our contemporary futures? Through attention to themes of value, temporality, and crisis, and by knitting together the pathologising and pathologised gaze, I have argued that preppers amplify conditions of the present in such a way that brings standardised versions of the neoliberal security society under scrutiny. Prepping portends possible futures of our ultimate neoliberal scripting; where, through our absorption of ever‐greater degrees of neoliberal forms of skeletal autonomy, material improvisation, anticipatory valuation, and crisis attunement function as survival instincts that structure the everyday. In conclusion, I wish to re‐emphasise three concerns traced across this paper in the challenge that prepping presents to the insecurity of networked dependency amidst weakening infrastructure, the privatisation of speculative futures, and the false promise of universal state security.

First, the prepper's valorisation of alternative object potentiality and centring of skilful value extraction exposes an anxiety of material dependency, felt in the contradiction between the individual's restricted agency alongside the infrastructural weakening of the state. Their crisis, dismissed as far‐fetched concerns over zombie apocalypse, is instead far more troubling. It is one of basic metabolic needs – the provision of food, water, shelter – and the understanding that existing institutions are incapable or unwilling to secure its reproduction. And so preppers imagine and foreshadow a socio‐material context where these needs are met outside of networked infrastructure, a “shadow infrastructure” or atomised material geography of metabolic survival operating in the ruins – or extremities – of capitalism (Tsing, 2015; Wakefield, 2018). By inhabiting and projecting social contexts where state infrastructure and welfare support are being reduced or withdrawn, by merging the endemic and personal crises of daily life with imaginations of extensive catastrophe, preppers challenge the individualisation of security by pointing to its structural and pernicious components. This reveals the coupled failure of our material and moral infrastructure, the bridging of material and ontological insecurity (Mills, 2018).

Second, the figure of the prepper challenges the limits to public participation in the production of secure futures and the imagination of alternatives. On the one hand, publics are encouraged and conditioned to be resilient citizens in the face of future crises. On the other, that opening to difference and change is foreclosed, constraining future imaginations to within the boundaries of the continuation of the capitalist present. Baraitser characterises this tension as “living in the time of waiting for the event” (2017, p. 5), caught between immanent temporal discontinuity and endless temporal monotony. In defiance of this suspension of agency in the imagination and production of alternative futures, preppers express a desire to be active participants in future‐making (Mitchell, 2001), as anticipation entrepreneurs, as hackers, makers, and engineers of everyday survival (Wakefield, 2018). In some ways this is a reflection of how macro‐structures and markets organise their activities in this context through practices of securitisation, off‐setting, and risks‐analysis. The prepper, however, challenges the commodification and privatisation of future speculation, recuperating it from venture capitalists. Rather than “harmonising with exhaustion” (Berardi, 2011) within a state of contingent dependency, preppers manage and circulate a food buffer, explore the multiple materialities of everyday objects, push the boundaries of condoned future scenarios and learn to purify water and light a fire in a world seemingly beyond repair. For this sense of agency to be extracted from the neoliberal security society, perhaps the inevitable outcome and remaining place it can be performed is the devastated imagined landscapes of social and infrastructural collapse. We may not like preppers' future visions any more than we like those of capitalism's perpetual‐present. But by recuperating the agency of future temporalities, by finding empowerment, pleasure, and vitality in moving closer to their metabolic vulnerability, preppers remind us that perhaps, this is a present that is worth surviving.

But will *we* survive? Third, then, the preppers' gaze is uncomfortable as it is exclusionary, casting the majority of us the unprepared victims of future crisis. When the prepper is constructed as a symbol of selfishness for capitalising on their relative security in the present to extend that advantage into the future, it is as if this contravenes an unwritten rule that security operates as a form of universal protection. In this sorting of victims from survivors, however, the preppers' biopolitical gaze mirrors and exposes that of the neoliberal security society. The myth of the universality of threat and protection implicit in crisis and apocalypse dictates that we are all potential victims, ultimately “in it together,” but also that we can all, when needed, shelter under state protection (Swyngedouw, 2010). This eliminates the reality of financial, health, and structural constraints to preparedness, and the differentiated promise of response that the state extends to its citizens. The 30days30ways campaign urges us to make “small” preparations for potential crisis, as if we all have the means and resources to do so. It urges us to look at our local risk register for risks that may be geographically differentiated, but not socially, economically, or racially specific or gendered. We are advised in certain emergency situations to “shelter in place” and await rescue, as if the emergency services have been adequately funded and trained, as if housing has been maintained to the appropriate safety standards, as if concerns are listened to, as if all lives matter. There is a cultural history to this socio‐economic hierarchy of security provision and the abandonment of the state's responsibility to protect, traceable back through access to air raid shelters in the Second World War and admission to nuclear bunkers in the Cold War era (Bennett, 2017). However, the state production of vulnerability through austerity alongside individual responsibilisation for crisis readiness adds qualitatively new layers to existing security inequalities. The prepper begins from an understanding of their own exclusion from the myth of universal state security. As one comment by a disabled prepper on a social media site put it, “People like me would be left to die.” Preppers disrupt the neoliberal security state by acknowledging its hierarchy of the damned.

Is this the reflection of a new type of governmentality or the result of the erosion of the existing one? There is a risk that by reinforcing the translation of crises into individuated scenes of citizenly responsibility, prepping simply props up rather than challenges neoliberal processes and austerity. Hitching preppers to a politically regressive apocalyptic imagination and framing them as mavericks for sensing their own vulnerability to the absence of state protection, media representations of prepper pathology reinforce the fantasy of the neoliberal security state with its resilient security citizen. Rather than a pathological figure, however, the prepper emerges as an anticipatory, emblematic, frontier subjectivity, born out of social settings structured through and around infrastructural dependency, but in the context of the widespread weakening of institutional political frameworks, of stretched regulatory apparatuses, of fragile infrastructure, of failures to respond to capitalism's self‐made catastrophes. The prepper also stands for that which has been repressed in late modernity, emerging in a context of a crisis of representation, a yearning for authenticity, for resourcefulness, for personal re‐evaluation and meaning, for a hand in economic and cultural crafting (Mitchell, 2001). The prepper offers one, albeit flawed, example of how among poorly distributed abundance a subjectivity can be performed that imagines and adjusts to scarcity, where apathy, rather than action, is framed as an irrational response to the reality of our everywhere crises. Even within media representations of their pathology, there is something of the prepper's future‐present that allures yet alarms. “Are they paranoid and delusional?,” the disembodied narrator of *UK Preppers: Surviving Armageddon* (NGC, 2012) asks, “or is the rest of Britain mad, for not joining them?”

## Data Availability

Due to the ethically sensitive nature of the research, no interviewees consented to their data being shared.

## ENDNOTES

1 Currently, anthropogenic climate change scepticism and the dismissal of Brexit concerns as “Project Fear” by the UK prepping mainstream necessitates prefixes – “Brexit preppers” or “Climate Change preppers” – to distinguish these subgroups.

2 Operational security.

3 Being “the grey man” refers to the ability to blend in and not draw attention to yourself or your preps.

4 In the following account I have masked all identifying details of my interviewees except gender to prevent mutual identification, and have paraphrased quotes taken from social media sources, as agreed with participants.

5 One large online group distinguished by a greater emphasis on exploring post‐SHTF [Shit Hits The Fan] “what if” scenarios had, of 2,300 members in August 2018, a breakdown of 91.7% identifying as male, 7.7% identifying as female, with 35–44 the largest age category at 31.3% (25.1% 45–54; 22.7% 25–34). A second large online group with a more family‐orientated focus of 3,000 members reported 85.6% identifying as male. In contrast, of 377 responses to a survey circulated in a Brexit‐related prepping group, 90.5% identified as female, 8% identified as male, with 45–54 the largest age category at 32.9% (29.2% 35–44, 18.8% 55–64). 43.4% reported having a university education.

6 WROL – without rule of law.

7 This contrasts with academic accounts that frame prepping through bunker ownership (Garrett and Klinke, 2019). See also Clinton 2017 for an excellent discussion of the architectural metaphor of the American bunker.

8 Shit Hits The Fan.

9 Meal ready‐to‐eat, commonly army rations packs.

10 It could be argued, however, that rather than moving “out of circulation,” by buying in surplus preppers are creating hyper‐circulation. While small in comparison to the USA, where Aldousari (2015) and Mills (2018) have illustrated the huge economic market that prepping feeds, in the UK the most consumptive manifestation of prepping supports a much smaller industry. Yet, by slowly and steadily building up a food buffer well in advance, preppers argue that they help mitigate the impact of future crisis‐related panic‐buying.

11 The End Of The World As We Know It.

12 See Ginn (2015) for a reappraisal of the political potential of apocalypse.
